# Presence of circulating Her-2 reactive CD8 T cells is associated with a lower frequency of MDSCs and better survival in elderly breast cancer patients

**DOI:** 10.1186/2051-1426-2-S3-P151

**Published:** 2014-11-06

**Authors:** Jithendra Kini Bailur, Brigitte Gueckel, Graham Pawelec, Evelyna Derhovanessian

**Affiliations:** 1 Department of Internal Medicine II, Section for Transplantation Immunology and Immunohaematology, University Hospital, Tuebingen, Germany; 2 Radiology Clinic, Diagnostic and Interventional Radiology, University Hospital, Tuebingen, Germany

## 

Breast cancer is one of the most common cancers among women. The risk of breast cancer has increased dramatically recently and a higher number of elderly women are being diagnosed with the disease. Myeloid-derived suppressor cells (MDSCs) have been implicated in breast cancer prognosis. Their frequency has been associated with tumour burden and studies have shown a poor prognosis in cases of metastatic breast cancer. MDSCs are known to impair the proliferation of T cells and to promote T cell apoptosis. Associations between MDSC levels and immune responses of the host to tumour-associated antigens had not been investigated. Here, we have studied T cell responses to Her-2 antigens, as well as the frequency of Tregs and MDSCs, in 40 untreated breast cancer patients (65-87 years of age) at diagnosis. After a 12-day in vitro expansion of memory cells stimulated by Her-2-derived peptides, CD4+ T cells reactive to Her-2 were detected in 84% of patients by intracytoplasmic staining simultaneously for TNF, IFN-γ, IL-2, IL-5, IL-10 and IL-17. In contrast, only 47% of patients had Her-2-reactive CD8+ T cells. The patients who lacked a CD8 response tended to have higher frequencies of Lineage(neg) CD14+ HLA-DR(neg) cells (p = 0.09). Importantly, the 5-year survival rate of patients who mounted a CD8+ T cell response and had a lower frequency of this particular subset of MDSC was 100% compared to only 53% in patients without Her-2-reactive CD8+ T cells and with higher frequencies of MDSCs (p = 0.03). This survival advantage was also observed in non-metastatic patients, with only a 38% 5-year survival in patients without a CD8 response and high levels of MDSCs compared to 100% survival of those who had a CD8 response and a lower frequency of MDSCs (p = 0.016). Similarly, for Tregs, patients who lacked a CD8 response to Her-2 and had higher frequencies of CD4+ Foxp3+ CD127(low) CD25+ Tregs had only 50% survival when compared to the 100% survival of the patients who mounted a CD8 response and had lower frequency of Tregs (p = 0.03). Also, for activated CD4+ CD45RA(neg)Foxp3(hi) Tregs, a similar trend was observed with 57% survival in patients who lacked a CD8 response and had higher frequencies of activated Tregs compared to the 100% survival in patients with a CD8 response and lower frequencies of activated Tregs (p = 0.06). Our data thus demonstrate a negative role of MDSCs and Tregs in prognosis of breast cancer patients, which might be through dampening favorable immune responses to tumour-associated antigens.

